# 

**Published:** 1852-10

**Authors:** 


### AMERICAN JOURNAL

OF

### DENTAL SCIENCE.

EDITED BY

CHAPIN A. HARRIS, M. D., D. D. S.

PROFESSOR OF THE PRINCIPLES AND PRACTICE OF DENTAL SURGERY IN THE BALTIMORE COLLEGE; MEMBER OF THE AMERICAN MEDICAL ASSOCIATION, ETC. ETC.

AND

ALFRED A. BLANDY, M. D., D. D. S.

Vol. III.—New Series.—No. 1.

Surgeon General's Office
Washington, D.C.
21688

PHILADELPHIA:
LINDSAY & BLAKISTON.
BALTIMORE:—ARMSTRONG & BERRY.

JOHN W. WOODS, PRINTER, BALTIMORE.

1852.

W
1
AM 45 M

### TO READERS AND CORRESPONDENTS.

Communications intended for publication, and all letters relating to the business of the Journal, or containing remittances in money, should be directed to Drs. Harris and Blandy, Baltimore, Md.

### *Medical and Dental Journals Received.*

1. *The American Journal of the Medical Sciences.* Edited by Isaac Hays, M. D., Surgeon to Wills Hospital, &c., July, 1852. *In Exchange.*

2. *The New York Dental Recorder.* Edited by C. C. Allen, M. D., Dentist, July, August and September, 1852. *In Exchange.*

3. *The Dental Register of the West.* Edited by James Taylor, M. D., D. D. S. Published by order of the Mississippi Valley Association of Dental Surgeons. July, 1852. *In Exchangc.*

4. *The Dental News Letter.* July, 1852. *In Exchange.*

5. *The London Medical Gazette.* May and June, 1852. *In Exehange.*

6. *Boston Medical and Surgical Journal.* Edited by J. V. C. Smith, M. D. July, August and September, 1852. *In Exchange.*

7. *The British and Foreign Medico-Chirurgical Review.* July, 1852. *In Exchange.*

8. *The Ohio Medical and Surgical Journal.* Edited by Richard L. Howard, M. D. June and September, 1852. *In Exchange.*

9. *The New York Journal of Medicine and the Collateral Sciences.* Edited by S. S. Purple, M. D. September, 1852. *In Exchange.*

10. *The Medical Examiner.* Edited by F. G. Smith, M. D., and John Biddle, M. D. July, August and September, 1852. *In Exchange.*

11. *The Southern Medical and Surgical Journal.* Edited by J. P. Garvin, M D. July, August and September, 1852. *In Exchange.*

12. *The New York Medical Gazette, and Journal of Health.* Edited by D. M Reese, M. D., LLD. July, August and September, 1852. *In Exchange.*

### *To Readers and Correspondents.*

13. *The New Jersey Medical Reporter, and Transactions of the New Jersey Medical Society.* Edited by JOSEPH PARISH, M. D. August and September, 1852. *In Exchange.*

14. *The Western Lancet and Hospital Reporter.* Edited by L. M. LAWSON, M. D., and GEORGE MENDENHALL, M. D. Not received *In Exchange.*

15. *The North Western Medical and Surgical Journal.* Edited by J. EVANS, M. D., and E. G. MEEK, A. M., M. D. July, August and September, 1852. *In Exchange.*

16. *The Western Journal of Medicine and Surgery.* Edited by L. P. YANDELL, M. D., and T. S. BELL, M. D. July, August and September, 1852. *In Exchange.*

17. *The New Hampshire Journal of Medicine.* Edited by EDWARD H. PARKER, A. M., M. D. July, August and September, 1852. *In Exchange.*

18. *The Charleston Medical Journal aud Review.* Edited by D. J. CAIN, M. D., and F. PEYREL PORCHER, M. D. August and September, 1852. *In Exchange.*

19. *The Buffalo Medical Journal.* Edited by AUSTIN FLINT, M. D. July, August and September, 1852. *In Exchange.*

20. *The Provincial Medical aud Surgical Journal.* Edited by G. W. H. RANKIN, M. D., and J. H. WALSH, Esq. July and August, 1852. *In Exchange.*

21. *The London Lancet*, Journal of British and Foreign, Surgical and Chemical Sciences, Chemistry, Literature and News. Editor THOMAS WAKELEY, M. D., for London. Sub-Editors, J. H. BENNET, M. D., and T. WAKELEY, M. R. C. S. E. May, August and September, 1852. *In Exchange.*

22 *The Northern Lancet and Gazette of Legal Medicine.* A monthly Journal of Medicine and General Science, Criticism, and Medical Jurisprudence. Edited by HORACE NELSON, M. D., and FRANCIS J. D. AVIGNON, M. D. August and September, 1852. *In Exchange.*

23. *The Dublin Medical Press.* July, August and September, 1852. *In Exchange.*

☞ The article from W. W. H. T. was received too late for publication in the present number, it will appear in our next. We have also received articles from H. L. Burpee, J. L. Levison, and S. A. Saltonstal, which will be published in our next issue.

*New York Medical College Announcement. Session* 1852-'3.

We notice that Dr. C. C. Allen is appointed to lecture on Dental Pathology and Dental Surgery. We have no doubt that lectures such as Dr. Allen is capable of delivering upon the structure and diseases of the teeth will be very useful to students of medicine and physicians. If we understand the character of Dr. Allen's lectureship, he is not expected to instruct young men in practical dentistry so as to qualify them for practice. We would be glad if lectureships on dental physiology and pathology were attached to all medical colleges, if filled by men as competent as Dr. Allen.

*Annual Catalogue and Announcement of the Medical Department of St. Louis University.*

The school seems to be flourishing. Their class of last year numbered seventy-five.

*Second Annual Announcement of the New York College of Dental Surgery,* Syracuse.

We wish well to this Institution, not the less so that we perceive the name of one of the graduates of the Baltimore College in the list of the Faculty. We allude to Dr. Ehrick Parmly, who graduated with high honors at the commencement in March, 1851, and subsequently, has spent most of his time in Paris. Dr. Westcott, the originator of the school, andformerly a professor in the Baltimore College, is known to the dental profession as a scientific dentist, and eminently qualified to discharge the duties belonging to the position which he fills in the Institution.

